# Perspective of adults in Saudi Arabia toward complementary and alternative medicine use for autism spectrum disorder: a cross-sectional study

**DOI:** 10.1186/s12906-023-04293-1

**Published:** 2023-12-13

**Authors:** Ghaidaa F. Khalifa, Bakriah Y. Alzubaidi, Dina A. Bamarouf, Yazeed B. Alsaedi, Omar H. Alayyafi, Majed M. Ramadan

**Affiliations:** 1 College of Applied Medical Sciences, King Saud bin Abdulaziz University for Health Sciences, Jeddah, Saudi Arabia; 2 King Abdullah International Medical Research Center, Jeddah, Saudi Arabia; 3 Ministry of National Guard, Health Affairs, Jeddah, Saudi Arabia

**Keywords:** Autism spectrum disorder, Complementary and alternative medicine, Perspectives, Saudi Arabia

## Abstract

**Background:**

Autism Spectrum Disorder (ASD) is a high-prevalence neurodevelopmental disorder characterized by communicational, social, and behavioral challenges. Complementary and alternative medicine (CAM) is a group of practices and products that fall outside the realm of conventional medicine practiced worldwide. Traditional CAM is a health practice that comes from a particular culture, such as the use of Zamzam water and black seeds in Saudi Arabia. CAM comprises widely utilized practices in Saudi Arabia for children of various ages and adults. In many cases, CAM is used to treat ASD. The aim of this study is to investigate the perspectives of adults in Saudi Arabia toward CAM for treating ASD.

**Methods:**

A cross-sectional study was conducted by distributing an online questionnaire to adults in different regions of Saudi Arabia.

**Results:**

A total of 4,311 adults participated in this study; 66.8% were females and 33.2% were male. Half of the participants reported that they knew about ASD (54.7%). More than half of the participants indicated that CAM—including art therapy (69.0%), physical exercise (67.0%), and limiting the use of electronic (55.4%)—could help in ASD management. Around half of the respondents indicated a belief that traditional treatment cannot help in treating ASD (53.9%). Only 20.0% of the respondents thought that ASD could be entirely treated using CAM therapies. The most common source of knowledge about CAM was social media and the internet (58.6%).

**Conclusions:**

The majority of participants in this study did not believe that ASD could be treated entirely using CAM. In addition, fewer than half of the participants believed that CAM therapies could improve different aspects of ASD, such as behavioral issues. The study highlighted the need for awareness among residents of Saudi Arabia regarding specific treatments for ASD.

## Background

Autism Spectrum Disorder (ASD) is a neurodevelopmental disorder characterized by communicational, social, and behavioral challenges [1]. ASD can also be associated with other conditions, including anxiety, depression, and attention deficit hyperactivity disorder (ADHD) [1]. Although the cause of ASD is unknown, multiple risk factors, such as environmental, maternal age above 40, excessive body weight, hypertension, and infection during pregnancy, are believed to play a role in the disorder [2]. ASD’s worldwide prevalence is estimated to be about one in 100 children [3]. In the Kingdom of Saudi Arabia (KSA), the number of children diagnosed with ASD has increased in recent decades [4]. A 2022 study conducted in Riyadh city found that the prevalence of ASD in KSA was estimated to be one in 40 children, which is close to recent trends in international studies [5].

In 2013, the American Psychiatric Association replaced the ASD subtypes in the previous version of its Diagnostics and Statistical Manual of Mental Disorders, which were autistic disorder, pervasive developmental disorder, and Asperger’s disorder [6]. The Diagnostic and Statistical Manual of Mental Disorders Fifth Edition (DSM-5), the current edition of the manual, bases ASD diagnosis on two domain issues: social communication and confined and repetitive sensory activities [6]. ASD criteria in DSM-5 state the child must have three persistent deficiencies in the social communication and interaction domain and at least two behaviors in the confined and repetitive sensory activities domain [1, 6]. In Saudi Arabia, after confirmation of an ASD diagnosis, treatment may be delayed due to geographic distance, annual income, parents’ understanding of ASD, and parents’ educational level [7]. A 2022 study concluded that community knowledge about ASD in Saudi Arabia is moderate to high [8]. Adults and children with ASD share common features. However, ASD patients may require a minor to a significant amount of caregiver support, depending on the severity of the disorder, which may lead parents to try complementary and alternative medicine (CAM) as a treatment approach for their child [1].

CAM comprises widely utilized practices in Saudi Arabia for different ages of children and adults, according to a 2017 study [9]. CAM is a group of diverse medical and healthcare systems, practices, and products that are not generally considered part of conventional medicine [10]. Traditional CAM is a health practice that comes from a particular cultural heritage, and its forms vary widely across the country, such as Zamzam water and black seeds in Saudi Arabia [11]. Family, friends, and mass media are the sources of information for most CAM users [12]. In 2012, a study reported that parents used mostly cultural and informal interventions to heal their children with ASD; such interventions include diet programs and hyperbaric oxygen therapy [13]. Another study found that most parents used non-medical treatments for their children, followed by biomedical treatments and cultural and religious treatments [7]. Parents who use CAM themselves use CAM for their children at a rate six times higher than non-user parents [14].

Approximately 95% of children with ASD have tried some form of complementary and alternative medicine (CAM) [15]. However, many parents don’t tell their doctor about CAM options they’ve used. These CAM approaches may interfere with children’s well-being. Also, CAM use is very common is Saudi Arabia [7, 9, 13]. Therefore, it is important to understand the public’s perspective on these interventions on ASD. The aim of this study is to investigate the perspectives of adults in Saudi Arabia toward complementary alternative medicine for autism spectrum disorder. In doing so, the study will explore the community’s knowledge about non-medical treatments such as CAM for ASD.

## Method

### Study design

A cross-sectional study using an online survey was conducted in Saudi Arabia during September and October 2022.

### Study population and sampling procedure

All Saudi and non-Saudi individuals who are currently living in Saudi Arabia and aged 18 years and above formed the study population. Participants were excluded from the study if they were under 18 years old and did not know about ASD. The questionnaire was distributed through social media applications by using convenience sampling techniques; respondents provided consent before participating in the study.

### The questionnaire tool

The questionnaire was inspired by “Questionnaire about pattern of complementary and alternative medicine (CAM) use among stroke survivors in Jeddah, Saudi Arabia.” This questionnaire was divided into two sections. Section A: Demographic data included eight questions, and Section B: Stroke and its relation to CAM, included 14 questions [16]. Before using and modifying the questionnaire, we obtained permission from the author of the research. After receiving permission, we made some modifications to the original questionnaire to meet the objectives of this study: the layout and questions were adapted to apply to ASD rather than stroke. In Section A: Demographic data, the changes included three questions. In Section B: complementary and alternative medicine, only three questions from the original questionnaire were retained because the other questions did not apply to ASD or the objectives of this study. We added seven questions regarding CAM interventions used for treating ASD which include specialized food. Art therapy, exercise. Massage therapy, supplements, Hyperbaric Oxygen Therapy (HBOT), limiting electronic devices usage. These interventions were taken from different studies concerning common CAM interventions for ASD [9, 13, 17–19]. We added eight questions regarding the attitude and knowledge towards the impact of CAM on different aspects of ASD. Moreover, the initial version of the questionnaire used in this study was written in the Arabic language and consisted of four sections, starting with a cover page, demographic information, CAM’s use and role in treating ASD, and the beliefs and reasoning behind CAM use for treating ASD. Face validity was reached through experts in the field who evaluated and peer-reviewed the questionnaire. Five of the experts had master’s degree in occupational therapy and two had Doctor of philosophy in Rehabilitation Sciences. Pilot testing was conducted by distributing the questionnaire to 21 adults in Saudi Arabia. All the respondents confirmed that the questionnaire was clear, understandable, and easy to answer. For internal consistency/reliability, we used Cronbach’s alpha. We estimated Cronbach’s alpha of 0.7, and the calculated result was 0.879, which indicates good consistency and reliability.

### Statistical analysis

Data analysis was carried out using RStudio (R version 4.1.1). Frequencies and percentages were used to express categorical variables. Items with multiple selections were analyzed using a multiple-response analysis. We used Pearson’s chi-squared test to assess the factors associated with positive perceptions toward the impact of CAM on ASD as well as those associated with recommending CAM to help manage ASD patients. The significantly associated factors from univariate testing were subsequently used as independent variables in multivariate binary logistic regression models to investigate the independent predictors of the primary outcomes. The results were expressed as odds ratio (OR) and 95% confidence interval (95% CI). Statistical significance was considered at p < 0.05.

## Results

A total of 4,311 people responded to our survey and were included in this study; 62.5% of them had obtained a bachelor’s degree. 54.3% were aged 18 to 28 years, and 52.6% were singles. The majority of respondents (95.4%) were Saudis. Approximately two-thirds of the participants were females (66.8%). Residents of the Western and Central regions represented 30.5% and 24.4% of the participants, respectively. Of the participants, 54.7% reported that they knew about ASD, and 24.4% of them were living with or knew someone with ASD. The most common age categories of those ASD patients were 9 to 13 years (38.5%) and 3 to 8 years (34.9%, Table 1).

**Table 1:** Demographic characteristics of the participants

Parameter	Category	N (%)
Gender	Male	1,430 (33.2%)
	Female	2,881 (66.8%)
Age (years)	Between 18 and 28	2,343 (54.3%)
	Between 29 and 39	827 (19.2%)
	Between 40 and 49	734 (17.0%)
	Between 50 and 66	407 (9.4%)
Educational level	High school	899 (20.9%)
	Diploma	412 (9.6%)
	Bachelor’s degree	2,694 (62.5%)
	Master’s degree	221 (5.1%)
	Doctoral degree	85 (2.0%)
Nationality	Saudi	4,112 (95.4%)
	Non-Saudi	199 (4.6%)
Region	Eastern region	636 (14.8%)
	Western region	1,315 (30.5%)
	South region	964 (22.4%)
	North region	343 (8.0%)
	Central region	1,053 (24.4%)
Marital status	Single	2,267 (52.6%)
	Married	1,843 (42.8%)
	Divorced	146 (3.4%)
	Widowed	55 (1.3%)
Know about ASD	Yes	2,360 (54.7%)
Live with or know someone diagnosed with ASD	Yes	1,052 (24.4%)
If yes, what is his/her age?*	3 to 8	367 (34.9%)
	9 to 13	405 (38.5%)
	14 to 19	176 (16.7%)
	20 or above	104 (9.9%)

*Data is based on 1052 participants who were living or knew someone with ASD

ASD: autism spectrum disorder

**Fig. 1:**
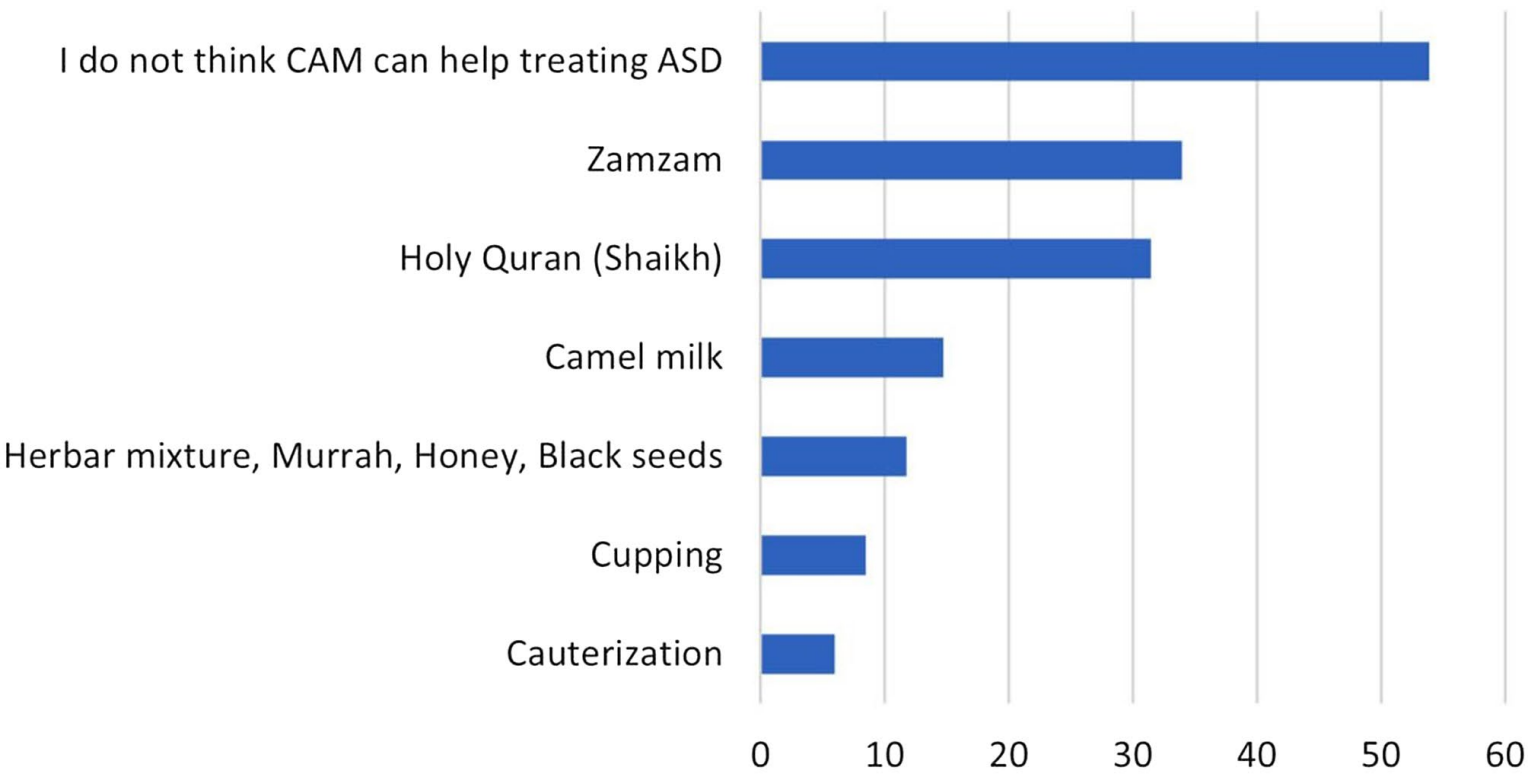
Participants’ responses regarding their perceptions of traditional CAM therapies for ASD

The 54.7% participants who reported that they knew about ASD indicated that these CAM therapies, including art therapy (69.0%), physical exercise (67.0%), and limiting the use of electronic devices by children with ASD (55.4%), could help in ASD management. Conversely, the most common approaches that fewer respondents saw as having therapeutic benefits for ASD patients were massage therapy (26.7%) and specialized types of food, such as gluten-free and casein-free diets (23.6%, Table 2). Additionally, 53.9% of the respondents indicated a belief that the traditional CAM therapies cannot help in treating ASD. Among the remaining participants, the most commonly perceived traditional therapies seen as possibly assisting in ASD management included Zamzam water (34.0%) and the Holy Quran (31.5%, Fig. 1**).** An analysis of the internal consistency of the CAM roles in ASD management (7 items) showed that the domain’s consistency was acceptable (Cronbach’s alpha = 0.741).

**Table 2:** The perceived roles of CAM in ASD management

Parameter	Category	N (%)
Specialized food such as gluten-free and casein-free diet can help in treating ASD	No	1,017 (23.6%)
	Yes	1,254 (29.1%)
	Do not know	2,040 (47.3%)
Art therapy (such as drawing) can help in treating ASD	No	403 (9.3%)
	Yes	2,975 (69.0%)
	Do not know	933 (21.6%)
Exercises can help in treating ASD	No	452 (10.5%)
	Yes	2,888 (67.0%)
	Do not know	971 (22.5%)
Massage therapy can help in treating ASD	No	1,151 (26.7%)
	Yes	1,419 (32.9%)
	Do not know	1,741 (40.4%)
Supplements such as omega 3 and vitamin D can help in treating ASD	No	709 (16.4%)
	Yes	1,980 (45.9%)
	Do not know	1,622 (37.6%)
Hyperbaric oxygen therapy (HBOT) can help in treating ASD	No	929 (21.5%)
	Yes	796 (18.5%)
	Do not know	2,586 (60.0%)
Limiting electronic devices usage by children can help in treating or preventing ASD	No	903 (20.9%)
	Yes	2,388 (55.4%)
	Do not know	1,020 (23.7%)
Which of the following traditional CAM do you think can help in treating patients with ASD	Camel’s milk	633 (14.7%)
	Zamzam	1,466 (34.0%)
	Holy Quran (Shaikh)	1,360 (31.5%)
	Herbal mixture, Murrah, Honey, Black seeds	507 (11.8%)
	Cauterization	259 (6.0%)
	Cupping	367 (8.5%)
	I do not think these traditional CAM could help in treating ASD	2,323 (53.9%)

ASD: autism spectrum disorder

**Fig. 2:**
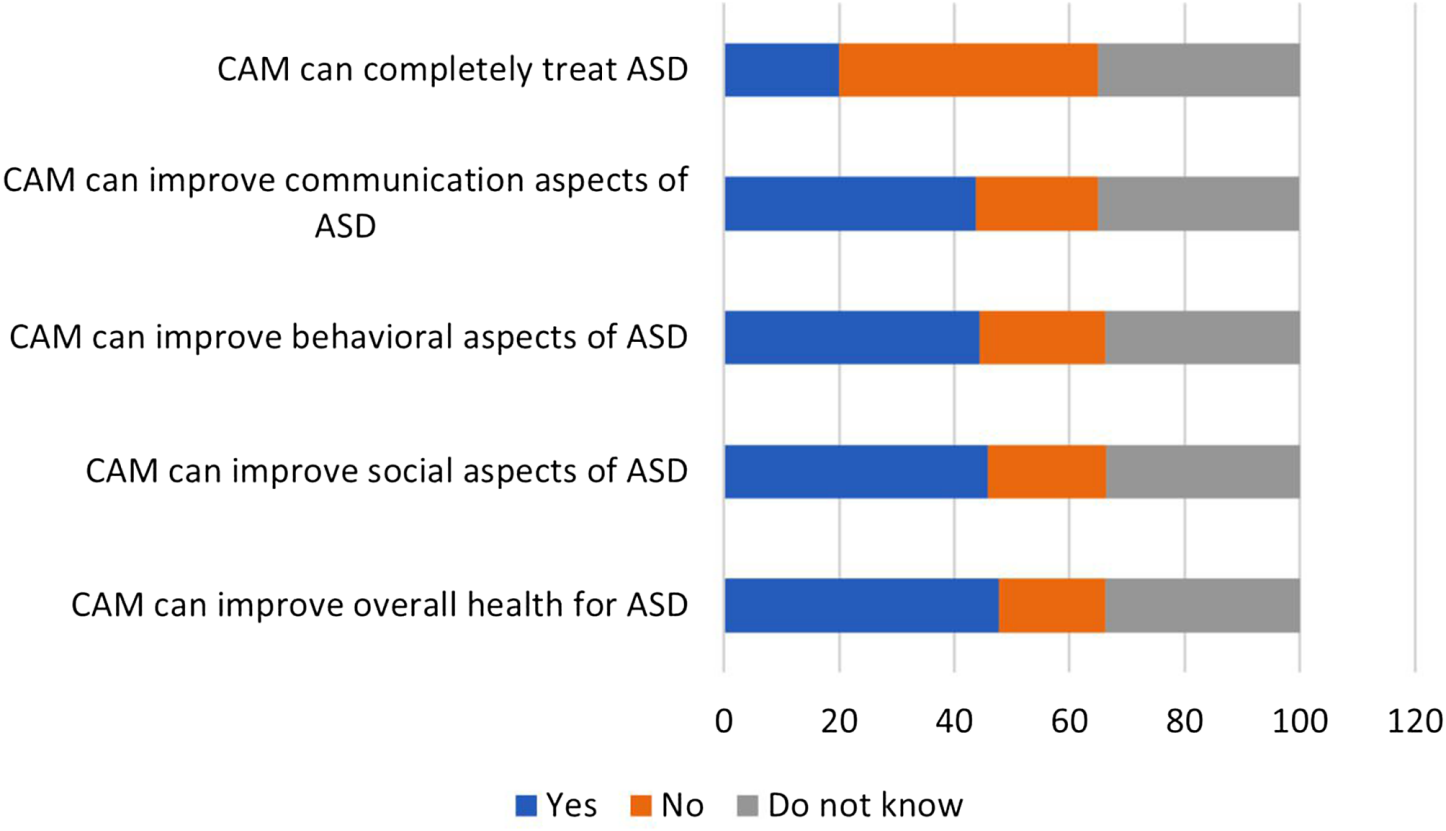
Participants’ attitudes toward the impact of CAM on different aspects of ASD

Fewer than half of participants believed that CAM could improve different aspects of ASD, including the communication aspects (43.7%), social aspects (45.9%), and behavioral aspects (44.4%), as well as the overall health of ASD patients (47.7%). Nevertheless, only 20.0% of respondents thought that CAM could completely treat ASD (Fig. 2). Of note: the majority of participants (79.4%) declared that they would ask a doctor before using CAM for ASD, whereas about one-third (38.7%) would recommend these traditional therapies for ASD. 40.2% might use CAM to treat ASD because it does not cause harm. The most frequently reported reasons for seeking out CAM are the lack of medical insurance (39.0%), the disabling nature of ASD (37.0%), and cultural beliefs (36.6%). Interestingly, the main sources of knowledge about CAM therapies for ASD include social media and the internet (58.6%) and friends and relatives (48.5%, Table 3). The internal consistency of the “attitudes” domain (8 items) was reliable (Cronbach’s alpha = 0.833) which is good.

**Table 3:** Participants’ attitudes and sources of knowledge about CAM therapies for ASD

Parameter	Category	N (%)
Do you think you should ask a doctor before using CAM?	No	413 (9.6%)
	Yes	3,425 (79.4%)
	Do not know	473 (11.0%)
The reason for using CAM is to treat ASD without causing harm	No	1,059 (24.6%)
	Yes	1,733 (40.2%)
	Do not know	1,519 (35.2%)
Would you recommend using CAM with ASD patients?	No	1,204 (27.9%)
	Yes	1,668 (38.7%)
	Do not know	1,439 (33.4%)
Reason(s) for seeking CAM*	No benefits from the medical treatments	1,313 (30.5%)
	Disabling nature of the disease	1,593 (37.0%)
	Difficult access to treatment	1,536 (35.6%)
	Strong beliefs in CAM	1,472 (34.1%)
	Cultural beliefs	1,578 (36.6%)
	No insurance	1,683 (39.0%)
	Evil eye, Fear of Magic (Jin)	918 (21.3%)
Main sources of knowledge about CAM for ASD patients*	Friend/relatives	2,092 (48.5%)
	Social media/internet	2,528 (58.6%)
	Health practitioners	943 (21.9%)
	CAM therapists	1,173 (27.2%)
	Families of other patients	1,143 (26.5%)
	Islamic scholars	471 (10.9%)
CAM can improve communication aspects of ASD	No	916 (21.2%)
	Yes	1885 (43.7%)
	Do not know	1510 (35.0%)
CAM can improve social aspects of ASD	No	882 (20.5%)
	Yes	1979 (45.9%)
	Do not know	1450 (33.6%)
CAM can improve behavioral aspects of ASD	No	939 (21.8%)
	Yes	1915 (44.4%)
	Do not know	1457 (33.8%)
CAM can improve overall health for ASD	No	801 (18.6%)
	Yes	2,055 (47.7%)
	Do not know	1,455 (33.8%)
CAM can completely treat ASD	No	1,936 (44.9%)
	Yes	862 (20.0%)
	Do not know	1,513 (35.1%)

*An asterisk indicates a multiple-response item

ASD: autism spectrum disorder; CAM: Complementary and Alternative Medicine

Results of the multivariate regression analysis showed that positive perceptions toward the role of CAM on ASD management were independently associated with the *female* gender (OR = 1.33, 95% CI, 1.16 to 1.53, p < 0.001), residing in the Central region (OR = 1.30, 95% CI, 1.05 to 1.60, p = 0.014), knowing about ASD (OR = 1.54, 95% CI, 1.34 to 1.76, p < 0.001), and having a bachelor’s degree (OR = 1.38, 95% CI, 1.18 to 1.62, p < 0.001) or a master’s degree (OR = 1.79, 95% CI, 1.31 to 2.45, p < 0.001). In contrast, participants who were less likely to experience positive perceptions toward CAM included those residing in the Northern region (OR = 0.73, 95% CI, 0.56 to 0.97, p = 0.028), in the older age categories, including between 29 and 39 years (OR = 0.47, 95% CI, 0.38 to 0.59, p < 0.001), between 40 and 49 years (OR = 0.36, 95% CI, 0.28 to 0.46, p < 0.001), and between 50 and 66 years (OR = 0.32, 95% CI, 0.24 to 0.43, p < 0.001, Table 4).

**Table 4:** Predictors of positive perceptions regarding the effects of CAM on ASD management

Parameter	Category	OR	95% CI	*p*-value
Gender	Male	—	—	
	Female	1.33	1.16, 1.53	**< 0.001**
Age	Between 18 and 28	—	—	
	Between 29 and 39	0.47	0.38, 0.59	**< 0.001**
	Between 40 and 49	0.36	0.28, 0.46	**< 0.001**
	Between 50 and 66	0.32	0.24, 0.43	**< 0.001**
Educational level	High school	—	—	
	Diploma	1.02	0.79, 1.30	0.888
	Bachelor’s degree	1.38	1.18, 1.62	**< 0.001**
	Master’s degree	1.79	1.31, 2.45	**< 0.001**
	Doctoral degree	1.42	0.89, 2.26	0.138
Nationality	Saudi	—	—	
	Non-Saudi	0.80	0.59, 1.08	0.144
Region	Eastern region	—	—	
	Western region	1.05	0.86, 1.28	0.628
	South region	0.96	0.78, 1.19	0.718
	North region	0.73	0.56, 0.97	**0.028**
	Central region	1.30	1.05, 1.60	**0.014**
Marital status	Single	—	—	
	Married	1.03	0.84, 1.28	0.764
	Divorced	0.91	0.62, 1.34	0.640
	Widowed	0.64	0.34, 1.18	0.160
Know about ASD	No	—	—	
	Yes	1.54	1.34, 1.76	**< 0.001**
Live with or know someone diagnosed with ASD	No	—	—	
	Yes	0.88	0.76, 1.03	0.109

ASD: autism spectrum disorder; CI: confidence interval; OR: odds ratio

The multivariate analysis revealed that being married (OR = 1.34, 95% CI, 1.08 to 1.65, p = 0.007) and knowing about ASD (OR = 1.15, 95% CI, 1.01 to 1.32, p = 0.040) were independent predictors of recommending CAM to help manage ASD (Table 5).

**Table 5:** Predictors of recommending CAM to help manage ASD

Parameter	Category	OR	95% CI	*p*-value
Gender	Male	—	—	
	Female	0.96	0.84, 1.11	0.616
Age	Between 18 and 28	—	—	
	Between 29 and 39	1.28	0.72, 1.59	0.332
	Between 40 and 49	1.86	0.91, 2.38	0.087
	Between 50 and 66	0.92	0.54, 1.70	0.987
Educational level	High school	—	—	
	Diploma	0.90	0.70, 1.15	0.382
	Bachelor’s degree	0.95	0.81, 1.12	0.552
	Master’s degree	0.78	0.57, 1.06	0.118
	Doctoral degree	0.99	0.62, 1.57	0.966
Nationality	Saudi	—	—	
	Non-Saudi	1.09	0.80, 1.46	0.591
Region	Eastern region	—	—	
	Western region	1.11	0.91, 1.36	0.304
	South region	1.03	0.83, 1.28	0.774
	North region	1.26	0.95, 1.66	0.102
	Central region	0.98	0.80, 1.21	0.846
Marital status	Single	—	—	
	Married	1.34	1.08, 1.65	**0.007**
	Divorced	0.87	0.59, 1.28	0.477
	Widowed	0.94	0.52, 1.69	0.849
Know about ASD	No	—	—	
	Yes	1.15	1.01, 1.32	**0.040**
Live with or know someone diagnosed with ASD	No	—	—	
	Yes	1.06	0.91, 1.24	0.439

ASD: autism spectrum disorder; CAM: Complementary and Alternative Medicine; OR: odds ratio; CI: confidence interval

## Discussion

This cross-sectional study explored the views of Saudi adults toward CAM that is used for ASD. A total of 4,311 adults from five regions of KSA participated in this study. The majority of the participants were female, and half of participants were between 18 and 28 years old. Most were Saudi and from the Western and Central regions. Around half of the respondents reported knowing about ASD. This is similar to other research reporting that Saudis’ knowledge about ASD was moderate, which indicates an increase in awareness of ASD [13].

In this study, more than half of the participants indicated that CAM therapies, including art therapy, physical exercise, and limiting the use of electronic devices, could help in the management of individuals with ASD. This is similar to a study reporting that mind-body interventions, such as art therapy, were leading modalities in adults with ASD but not in children and adolescents [17]. Conversely, the most common therapeutic approaches that respondents saw as perhaps having no benefits for ASD were massage therapy and specialized types of food, such as gluten-free and casein-free diets. However, this contrasts with studies indicating massage therapy and special diets were some of the most used modalities for children with ASD [13, 17–19]. In addition, almost half of the participants reported a belief that traditional treatments cannot help with ASD. The remaining half indicated that the most common CAM therapies that may help in ASD treatment are Zamzam water and the Holy Quran. This concurs with a qualitative study where parents reported the most frequent interventions to help children with ASD were cultural interventions, including reading Al-Quran [13]. Also, a systematic review showed that the most commonly used CAM therapies in KSA were reciting the Quran, prayer, and reciting the Quran on Zamzam water [9]. This may be related to the conservative and religious traditions and customs of the Saudi community.

In this study, a minority of participants believed that CAM could improve the symptoms of ASD and its use would not cause harm. Also, only a few believed that it could completely heal ASD. Similarly, most of the parents in a qualitative study initially provided their children with ASD complementary health approaches (CHAs) because they believed they were safe and could improve the symptoms of ASD and, in a few cases, completely cure the disorder [17]. The majority of participants believed that they should consult a doctor before using CAM. This suggests that most of the participants from KSA are aware enough not to use CAM interventions without an appropriate consultation. A few studies reported that almost half of CAM users consulted with or disclosed to physicians about the use of CAM [19–21]. Unwillingness to stop using CAM was one of the most common reasons for non-disclosure [21]. Another study showed that most of the participants believed that CAM should not be used without a medical consultation [22]. In this study, the most frequent reasons for resorting to CAM were the lack of medical insurance, the disabling nature of ASD, and cultural beliefs. However, a systematic review indicated that the most common reasons for seeking CAM were uselessness of medical intervention, success of CAM use, personal preferences, and health services problems [9]. Interestingly, the sources of knowledge about CAM among the population of this study were mainly social media and the internet, followed by friends and relatives. This is predictable since the social network is a primary source of information, and people mostly trust what they learn from their family members. Similarly, a couple of studies reported that friends and family members were sources of information about CAM for ASD and other conditions [21, 22].

This study’s multivariate regression analysis showed that positive perceptions toward the role of CAM on ASD management were independently associated with the female gender, living in the Central region, knowing about ASD, and having a bachelor’s or master’s degree. This is consistent with other studies showing females were more likely to use CAM [21–25]. Also, other studies have shown that higher education might be associated with CAM use [18, 21]. Nevertheless, in one qualitative study, the education level of parents was not associated with use of CHA for their children [17].

### Strengths and limitations

To our knowledge, this study is the first survey in Saudi Arabia to investigate the attitudes of adults in Saudi Arabia towards CAM usage for ASD. Nevertheless, in this study, most of the participants are from the Central region which may affect the generalizability of the results. Because this study used a nonprobability sampling technique, convenience sampling may have influenced the generalizability of our findings. Most of the participants were under the age of 28 years old which also could have affected the generalizability of the study. Data collection via platform may have missed some of the intended demographic groups. Our newly constructed questionnaire tool (not standardized) was created from different instruments and tested on a small group of people from the general public.

### Implications

Our results suggest the importance of increasing awareness among families of individuals with ASD to not rely on social media as a primary source for intervention. Also, healthcare providers need to take into consideration the possible use of CAMs by families of children with ASD. Healthcar providers are encouraged to ask questions and have discussions with families. Practitioners should inform the families about the benefits and harms of CAMs and their possible interactions with prescribed interventions. These information should be shared with the public as well since relatives and friends were one of the main sources of information.

### Future studies

There are different cultures in KSA, so a future qualitative study will assist in finding other kinds of CAM that can help researchers analyze more deeply. Furthermore, in such a future study, more options should be added to pinpoint the circumstances that lead families to use CAM, and the inclusion criteria should be changed to only families or relatives of individuals with ASD who have used CAM.

## Conclusion

This investigative study demonstrated the viewpoint of Saudi adults on CAM with ASD. The study found that most participants think that some forms of CAM may help with ASD, but not the traditional CAM. However, few of the participants believe that CAM can totally treat ASD. Fewer than half of Saudi adults surveyed believed that CAM can improve different aspects of ASD, such as the behavioral aspect. The study highlighted the need for awareness among residents of Saudi Arabia regarding specific treatments for ASD.

## Data Availability

The datasets used and/or analyzed during the current study are available from the corresponding author upon reasonable request.

## References

[1] Centers for Disease Control and Prevention. Autism spectrum disorder (asd). https://www.cdc.gov/ncbddd/autism/facts.html. Accessed 26 Jan 2022.

[2] Lyall K, Ashwood P, Van de Water J, Hertz-Picciotto I (2014). Maternal immune-mediated conditions, autism spectrum disorders, and developmental delay. J Autism Dev Disord.

[3] World Health Organization: Autism. https://www.who.int/news-room/fact-sheets/detail/autism-spectrum-disorders#:~:text=The%20level%20of%20intellectual%20functioning,profound%20impairment%20to%20superior%20levels.&text=It%20is%20estimated%20that%20worldwide,prevalence%20varies%20substantially%20across%20studies.Accessed 28 Jan 2023.

[4] Alanazi AS. Autism spectrum disorder in Saudi Arabia: current issues and challenges. Arab J Sci Publishing (AJSP) ISSN 2663:5798.

[5] AlBatti TH, Alsaghan LB, Alsharif MF, Alharbi JS, BinOmair AI, Alghurair HA, Aleissa GA, Bashiri FA (2022). Prevalence of autism spectrum disorder among Saudi children between 2 and 4 years old in Riyadh. Asian J Psychiatry.

[6] Diagnostic (2013). Statistical Manual of Mental disorders (dsm-5).

[7] Alnemary FM, Aldhalaan HM, Simon-Cereijido G, Alnemary FM (2017). Services for children with autism in the Kingdom of Saudi Arabia. Autism.

[8] Abualhommos AK, Aldoukhi AH, Alyaseen AAA, AlQanbar FA, Alshawarib N, Almuhanna ZA (2022). Community knowledge about autism spectrum disorder in the Kingdom of Saudi Arabia. IJERPH.

[9] Alrowais NA, Alyousefi NA (2017). The prevalence extent of complementary and alternative medicine (CAM) use among saudis. Saudi Pharm J.

[10] National Center for Complementary and Integrative Health (NCCIH). US Department of Health and Human Services. https://www.nccih.nih.gov/. Accessed 16 Jan 2023.

[11] World Health Organization. WHO global report on traditional and complementary medicine 2019. https://www.who.int/publications/i/item/978924151536. Accessed 16 May 2019.

[12] Elolemy AT, AlBedah AMN (2012). Public knowledge, attitude and practice of complementary and alternative medicine in Riyadh Region, Saudi Arabia. Oman Med J.

[13] Alqahtani MM (2012). Understanding autism in Saudi Arabia: a qualitative analysis of the community and cultural context. J Pediatr Neurol.

[14] Gad A, Al-Faris E, Al-Rowais N, Al-Rukban M (2013). Use of complementary and alternative medicine for children: a parents’ perspective. Complement Ther Med.

[15] Bent S, Hendren RL (2015). Complementary and alternative treatments for autism part 1: evidence-supported treatments. AMA J Ethics.

[16] Alhawsawi T, Alghamdi M, Albaradei O, Zaher H, Balubaid W, Alotibi HA, Aboalshamat K, Alzahrani S (2020). Complementary and alternative medicine use among ischemic Stroke survivors in Jeddah, Saudi Arabia. Neurosciences J.

[17] Lindly OJ, Thorburn S, Heisler K, Reyes NM, Zuckerman KE (2018). Parents’ use of complementary health approaches for young children with autism spectrum disorder. J Autism Dev Disord.

[18] Höfer J, Hoffmann F, Bachmann C (2017). Use of complementary and alternative medicine in children and adolescents with autism spectrum disorder: a systematic review. Autism.

[19] Höfer J, Hoffmann F, Kamp-Becker I, Küpper C, Poustka L, Roepke S, Roessner V, Stroth S, Wolff N, Bachmann CJ (2019). Complementary and alternative medicine use in adults with autism spectrum disorder in Germany: results from a multi-center survey. BMC Psychiatry.

[20] Alnafia A, Binyousef FH, Algwaiz A, Almazyed A, Alduaylij T, Alolaiwi O, Alajlan A, Alsuhaibani M, Alenazi KA. Attitudes towards complementary and alternative medicine among pediatricians in Saudi Arabia. Cureus. 2021;13(12). 10.7759/cureus.20486 PMC8761483 35070536

[21] Farooqui M, Alreshidi H, Alkheraiji J, Abdulsalim S, Alshammari MS, Kassem L, Hussein S, Ismail WI. A cross-sectional assessment of complementary and alternative medicine (CAM) use among patients with chronic diseases (CDs) in Qassim, Saudi Arabia. Healthcare. 2022; 10.3390/healthcare10091728. 10.3390/healthcare10091728 PMC9498487 36141338

[22] Alazmi AS, Alhamad J (2020). Attitudes and practices of complementary and alternative medicine among patients attending primary care center in Saudi Arabia: a prospective cross-sectional study. J Family Med Prim Care.

[23] Jazieh AR, Al Sudairy R, Abulkhair O, Alaskar A, Al Safi F, Sheblaq N, Young S, Issa M, Tamim H (2012). Use of complementary and alternative medicine by patients with cancer in Saudi Arabia. J Altern Complement Med.

[24] Al-Zahim AA, Al-Malki NY, Al-Abdulkarim FM, Al-Sofayan SA, Abunab HA, Abdo AA (2013). Use of alternative medicine by Saudi Liver Disease patients attending a tertiary care center: prevalence and attitudes. Saudi J Gastroenterol.

[25] Thomson P, Jones J, Evans JM, Leslie SL (2012). Factors influencing the use of complementary and alternative medicine and whether patients inform their primary care physician. Complement Ther Med.

